# Genome-wide identification, characterization, and expression analysis of lineage-specific genes within zebrafish

**DOI:** 10.1186/1471-2164-14-65

**Published:** 2013-01-31

**Authors:** Liandong Yang, Ming Zou, Beide Fu, Shunping He

**Affiliations:** 1 The Key Laboratory of Aquatic Biodiversity and Conservation of Chinese Academy of Sciences, Institute of Hydrobiology, Chinese Academy of Sciences, Wuhan, Hubei 430072, People’s Republic of China; 2 University of Chinese Academy of Sciences, Beijing 100039, People’s Republic of China

**Keywords:** Teleost, Lineage-specific gene, Transcriptome, Zebrafish embryogenesis

## Abstract

**Background:**

The genomic basis of teleost phenotypic complexity remains obscure, despite increasing availability of genome and transcriptome sequence data. Fish-specific genome duplication cannot provide sufficient explanation for the morphological complexity of teleosts, considering the relatively large number of extinct basal ray-finned fishes.

**Results:**

In this study, we performed comparative genomic analysis to discover the Conserved Teleost-Specific Genes (CTSGs) and orphan genes within zebrafish and found that these two sets of lineage-specific genes may have played important roles during zebrafish embryogenesis. Lineage-specific genes within zebrafish share many of the characteristics of their counterparts in other species: shorter length, fewer exon numbers, higher GC content, and fewer of them have transcript support. Chromosomal location analysis indicated that neither the CTSGs nor the orphan genes were distributed evenly in the chromosomes of zebrafish. The significant enrichment of immunity proteins in CTSGs annotated by gene ontology (GO) or predicted *ab initio* may imply that defense against pathogens may be an important reason for the diversification of teleosts. The evolutionary origin of the lineage-specific genes was determined and a very high percentage of lineage-specific genes were generated via gene duplications. The temporal and spatial expression profile of lineage-specific genes obtained by expressed sequence tags (EST) and RNA-seq data revealed two novel properties: in addition to being highly tissue-preferred expression, lineage-specific genes are also highly temporally restricted, namely they are expressed in narrower time windows than evolutionarily conserved genes and are specifically enriched in later-stage embryos and early larval stages.

**Conclusions:**

Our study provides the first systematic identification of two different sets of lineage-specific genes within zebrafish and provides valuable information leading towards a better understanding of the molecular mechanisms of the genomic basis of teleost phenotypic complexity for future studies.

## Background

Teleosts, which roughly constitute 96% of all living fishes and half of the extant vertebrate, are the most phenotypically diversified and species-rich group of all the vertebrate species [1]. The vast morphological and species diversity of teleosts have received intense attention worldwide because of their importance in both scientific research and aquaculture. However, the genomic basis of the complex phenotype of teleosts during evolution remains obscure, despite the increasing amount of genome and transcriptome sequence data available. One important mechanism for the phenotypic diversity of species is the duplication of genes and entire genomes [2]. Evidence has recently been accumulated to allow a consensus to be reached that all teleosts experienced an additional whole genome duplication (fish-specific genome duplication, FSGD or 3R), which occurred after the basal ray-finned fishes separate from the actinopterygian stem lineage but before the teleosts began radiation [2-19]. Combining the absolute dates and phylogenetic timing of the 3R duplication, some groups have thought that the FSGD might be causally related to an increase in the number of species as well as their biological diversity [2,4,7,9,11-13,15,19-23]. However, if the FSGD (3R) was responsible for the evolutionary success and astounding biological diversification of teleosts, it must have occurred prior to the radiation of teleosts. With the fossil record, paleontological evidence have suggested that the first appearance of most of the extant teleosts was only about 235 million years ago [24,25], which is shorter than the FSGD that occurred at least 320 million years ago. Thus, it would be inappropriate to think that the FSGD was a major driving force behind the rapid radiation of teleosts [3,4,11,12,19,20]. Furthermore, considering the large amount of fossil data for basal ray-finned fishes, a consequential FSGD would not provide sufficient explanation for the morphological complexity of teleosts [26].

Besides the fish-specific genome duplication, alternative explanations for the increasing morphological complexity of teleosts include their experience with a higher rate of chromosomal rearrangements [27,28] and a faster evolution of protein sequences [29] and conserved noncoding elements (CNEs) [30] compared to cartilaginous fishes and mammals. Their implications for the evolution and diversity of teleosts have been intensively discussed [28]. However, conserved teleost lineage-specific genes have been poorly characterized.

Lineage-specific genes, also referred to as taxonomically restricted genes (TRGs) [31] are defined as genes found in one particular taxonomic group but share no sequence similarity with genes from other lineages [31-37]. With the advent of large-scale genome sequencing projects for a wide range of species, lineage-specific genes have been extensively studied in mammals [34,38,39], insects [33,40-42], plants [36,43-45], and microbial species [46-50]. Lineage-specific genes are a significantly abundant component of all genomes sequenced to-date [31], which defies an early hypothesis that an increasing database size would eventually reduce the number of lineage-specific genes [51]. Orphan genes were first discussed when analyzing the yeast genome; approximately one-third of the identified genes fell into this category [51,52]. Likewise in *Drosophila melanogaster,* the most accurate and complete genome analyzed, lineage-specific genes were found to make up nearly 18.6% of the total genes [53]. Apart from being abundant, lineage-specific genes have also been thought to be important for lineage specific traits and adaptations [54]. In *Hydra*, for example, interspecific differences in tentacle formation are closely related to the changes in the expression of taxonomically restricted genes [55]. And in *Drosophila*, the *flightin* gene is specifically important for increasing the frequency of the flight muscle to deliver the maximum power to the wing, which is a rather specific adaptation for the Dipterans [33]. Although abundant in quantity and important in functionality, the evolutionary origin of lineage-specific genes is still enigmatic. Several hypotheses about the origin of lineage-specific genes, including gene duplication followed by rapid sequence divergence, lateral gene transfer, accelerated evolutionary rate, artifacts from genome annotation, as well as de novo evolution from noncoding sequences have been proposed [36]. Despite the fact that the origin and evolution of lineage-specific genes is still poorly understood, the identification, characterization, function, and expression analysis of lineage-specific genes may provide a better understand for lineage-specific adaptation, such as the successful diversification of teleosts.

In this study, we identified Conserved Teleost-Specific Genes (CTSGs) and orphan genes in zebrafish using comparative genomics. We then characterized each set of these genes by diverse features, including gene size, protein size, exon number, GC content, transcript support, and chromosomal locations. As a large portion of the CTSGs and orphan genes have no known function, *ab initio* predictions using ProtFun were performed to infer possible biological functions. We then explored the evolutionary origin of lineage-specific genes and performed a comprehensive analysis of their tissues and developmental stages specific expression patterns using the wealth of available expression data, including EST and RNA-seq data, which in turn provided important complementary datasets that may be used to uncover their functions in the future. Collectively, identification of lineage-specific genes as well as orphan genes and future studies of their function by means of target gene knockdown [56] or knockout [57,58] will no doubt help increase our understanding of the molecular basis of the successful diversification of teleosts.

## Results

### Identification of CTSGs and orphan genes

**Figure 1:**
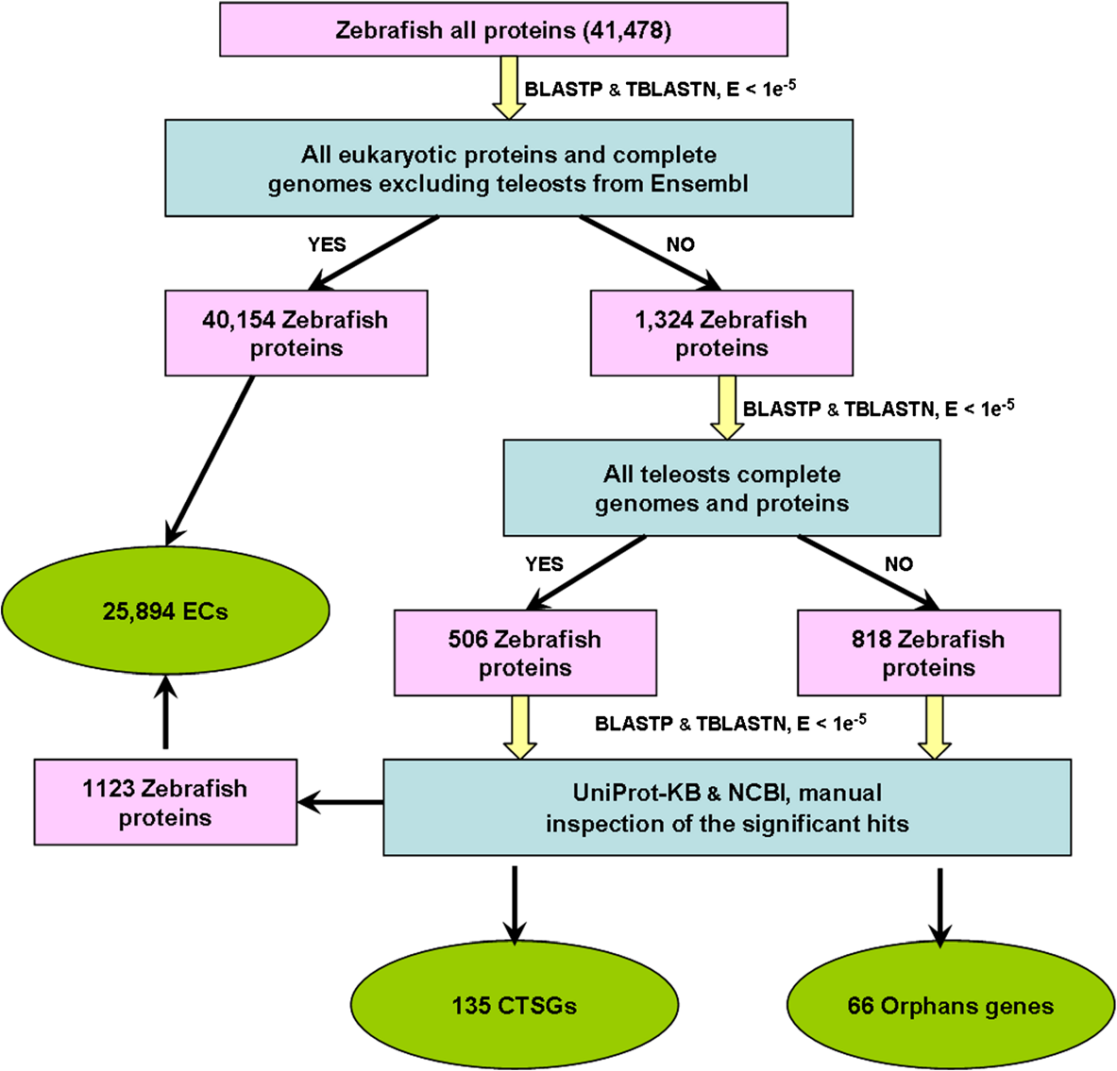
Procedure for identifying lineage-specific genes in zebrafish.

The procedure used to identify orphan genes and CTSGs was modified from previous studies [32-34,36,39-41,43-45,50,59-61]. We used the blast-Basic Local Alignment Search Tool [62] with default parameters for all assignments. Firstly, we used BLASTp to search for homologs of all 41,478 proteins annotated of zebrafish in each of the other 52 species-levels outside the teleostei protein sets with an e-value cutoff of 10^-5^. Those proteins for which we could not find any homolog in any alternative-spliced forms were used for the next set of searches, which was done using tBLASTn to search for homologs against the genome of the same species. In this step of the search, we only performed the tBLASTn search when there were no annotated proteins of the genome of the species, such as the genomes of elephant shark and *Salmo salar*. A total of 40,154 *Danio rerio* proteins with significant sequence similarity (E-value < 1e^-5^) to either a protein or genomic sequence from a species outside the group of teleosts were defined as the evolutionarily conserved proteins (Figure 1). Secondly, the remaining 1,324 *D. rerio* proteins with no significant similarity to any sequence (protein or genomic) outside the group of teleosts were further searched against the proteins and the genome of the group of teleosts, including *Oryzias latipes, Gasterosteus aculeatus, Takifugu rubripes, Tetraodon nigroviridis, Oreochromis niloticus, Gadus morhua, Salmo salar*, using BLASTp and tBLASTn with E-values the same as the first step. This step produced two datasets: 1) 506 proteins with significant sequence similarity only to the sequences from the group of teleosts and 2) 818 proteins that had no significant sequence similarity to any sequences within the species we used in this study (Figure 1). In order to further eliminate false positives due to the incompleteness of the annotated protein sets and genomes, these two sets of proteins were then searched against the UniProt Knowledgebase (UniProtKB) and the current non redundant protein database in NCBI using BLASTp. After manual inspection of the alignments, several genes initially assigned to the taxonomic categories were moved to the correct classification. Finally, the protein ID of evolutionarily conserved proteins were transformed to their coding genes’ ID and the final sets of CTSGs, orphan genes, and Evolutionarily Conserved genes (ECs) contained 135, 66, and 25,894 *D. rerio* genes, respectively (Figure 1, Additional file 1: Table S2, Additional file 2: Table S3).

### Characterization of CTSGs and orphan genes

Several general tendencies of lineage-specific genes and orphan genes (similar to older genes and younger genes) in different taxa have been observed in previous studies. First, younger genes tend to have shorter gene size, shorter proteins, and fewer exon numbers than older ones [36,40,43-45,63,64]. Second, younger genes show lower expression on average [40,63,65,66]. To determine whether this was the case for the two sets of lineage-specific genes within zebrafish, we characterized and compared the genic properties of the CTSGs and orphan genes with those of the Evolutionarily Conserved gene sets (Table 1). Just like the orphan genes in other species, such as primates, insects, and plants, the average gene size, average protein size, and average exon numbers per gene of the orphan genes were significantly lower than that of the CTSGs and ECs gene sets (one-way ANOVA; *p* < 0.01). However, the GC content of the orphan genes was significantly higher than that of the CTSGs and the ECs gene sets (one-way ANOVA; *p* < 0.01), with the CTSGs having the lowest GC content. The observation that the CTSGs had the lowest GC content was consistent with several previous studies, specifically those about lower GC content of taxonomically-restricted genes in Drosophila [33], honey bee [40], and *Arabidopsis thaliana*[36,43]. Meanwhile, the observation that the orphan genes within zebrafish had the highest GC content was consistent with the elevated GC content within the coding sequences of the lineage-specific genes in rice [44]. Overall, the genic features of the two sets of lineage-specific genes within zebrafish were distinct from the evolutionarily conserved gene sets and were comparable to their counterparts in other species.

**Table 1:** Gene characteristics of lineage-specific genes

	**Gene Size (nt)**	**Protein Size (aa)**	**Exon Number**	**GC Content**	**Transcripts**
	**mean ± SE**	**mean ± SE**	**mean ± SE**	**mean ± SE**	**%**
Orphan genes	1055.41 ± 117.51	131.27 ± 15.81	2.29 ± 0.13	40.63 ± 1.15	36.36
CTSGs	13786.82 ± 11739.11	167.62 ± 7.44	3.65 ± 0.13	36.87 ± 0.40	64.44
ECs	27763.23 ± 289.38	497.86 ± 2.64	8.93 ± 0.04	37.67 ± 0.03	81.14

**Figure 2:**
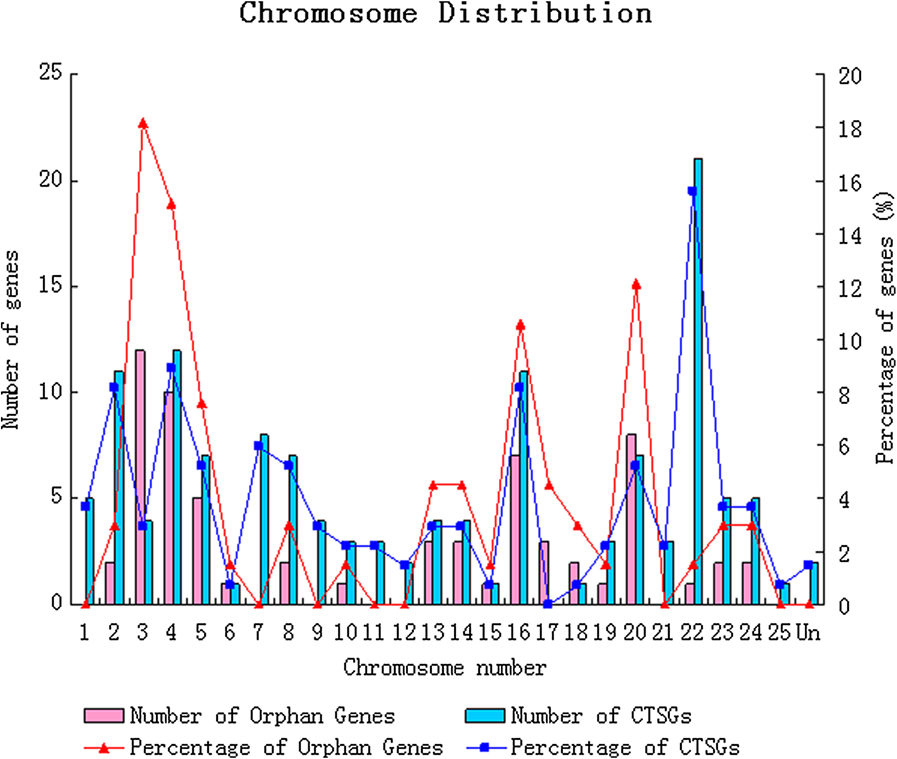
The numbers of lineage-specific genes on each chromosome in zebrafish. Both numbers and percentages are shown.

The physical locations of the two sets of lineage-specific genes within zebrafish were assigned to the 25 zebrafish chromosomes according to the information from Ensembl version 64 [67]. The number as well as the percentage of lineage-specific genes on each chromosome was also counted. Neither the CTSGs nor the orphan genes were distributed evenly within the different chromosomes of zebrafish (Figure 2). For the orphan genes, four chromosomes (3, 4, 16, and 20) harbored a total of 37 orphan genes, each with seven to twelve orphan genes, which is more than fifty percent of the total number of orphan genes in zebrafish. However, there were seven chromosomes (1, 7, 9, 11, 12, 21, and 25) which harbored no orphan genes. The chromosomal distribution of the CTSGs was basically similar to that of the orphan genes. Approximately fifty percent of CTSGs (45.9%) were located within five chromosomes (2, 4, 16, 20, and 22), whereas five chromosomes (6, 15, 17, 18, and 25) each housed only one or no CTSGs. In order to test whether the percentage of lineage-specific genes on each of chromosome correlated with the length of the chromosomes in zebrafish, Spearman’s test was employed. Spearman’s test showed that the number of CTSGs on each chromosome significantly correlated with the length of their respective chromosome (*p* = 0.019, *r* = 0.448), whereas the number of orphan genes on each chromosome did not correlate with the length of their respective chromosome (*p* = 0.058, *r* = 0.369).

Our results showed a trend similar to previous studies that found that younger genes exhibited lower expression on average [32]. To determine whether a gene had evidence of expression from EST or full-length cDNA (FL-cDNA), we first downloaded the unigene data using BIOMART. If a gene model of zebrafish was annotated with an EST or FL-cDNA in ENSEMBL, then we considered that gene to have transcript support. Combining these two sets of results, the percentage of genes with transcript support was determined (Table 1). The transcript support for orphan genes (36.4%) and for the CTSGs (64.4%) was significantly lower than that for EC genes (81.1%), with the orphan genes having the lowest transcript support (one-way ANOVA, *p* < 0.01).

### Functional inference using ProtFun

It has been shown that few of the highly taxonomically restricted genes (lineage-specific genes) have been the focus of experimental work or could be characterized by GO categories [40,49,61]. In order to determine whether this is the case for the two sets of lineage-specific genes within zebrafish, the function annotations of CTSGs and orphan genes available at Ensembl [67] were explored using Biomart [68]. As expected, a significantly large percentage (34.8%) of orphan genes were annotated as uncharacterized proteins (31.8%) or had no description information (3%) and there was only one gene annotated with GO term accession. Likewise, about 34.1% of CTSGs were annotated as uncharacterized proteins and hypothetical proteins or even have no description information and approximately 68.9% of CTSGs were without GO term annotations. These observations suggest that the functions of most of the two sets of lineage-specific genes are unknown and some of the annotated genes may be the result of incorrect annotations, considering that there were seven orphan genes encoding proteins less than ten amino acids. With respect to the remaining orphan genes with functional annotations in Ensembl, there was no bias seen in specific functions; however, there were about eight genes of CTSGs involved in immunity, indicating that immune response may be very important in the radiation of teleosts.

**Figure 3:**
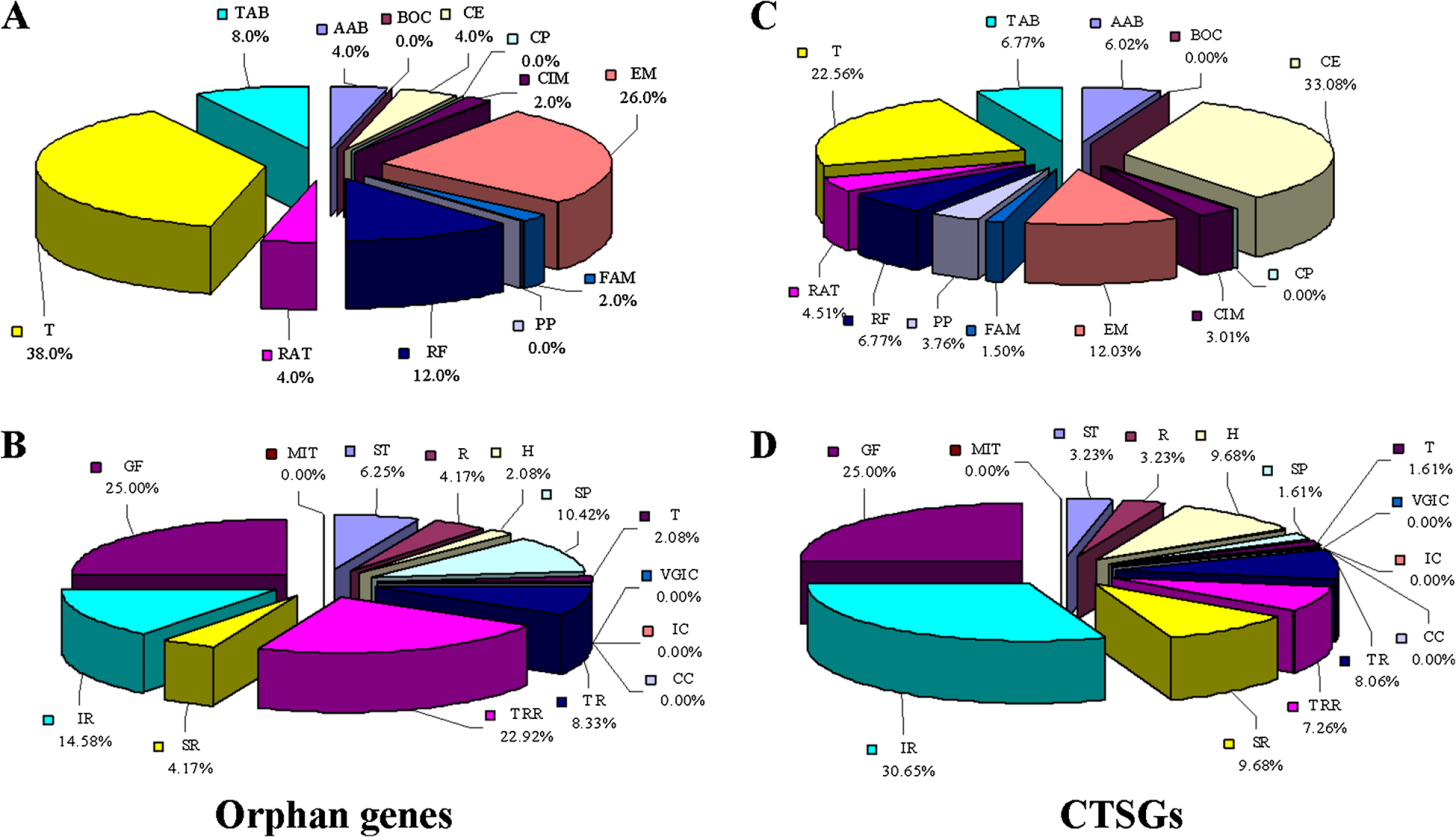
**Functional categorization of lineage-specific genes within zebrafish.** Cellular role (**A, C**) and GO category (**B, D**) were determined *ab initio* for orphan genes (**A, B**) and CTSGs (**C, D**) by ProtFun and percentage of genes included in each category are given. Cellular role categories are: AAB-amino acid biosynthesis, BOC-biosynthesis of cofactors, CE-cell envelope, CP-cellular processes, CIM-central intermediary metabolism, EM-energy metabolism, FAM-fatty acid metabolism, PP-purines and pyrimidines, RF-regulatory functions, RAT-replication and transcription, T-translation, TAB-transport and binding. GO categories are: ST-signal transducer, R-receptor, H-hormone, SP-structural protein, T-transporter, IC-ion channel, VGIC-voltage-gated ion channel, CC-cation channel, TR-transcription, TRR-transcription regulation, SR-stress response, IR-immune response, GF-growth factor, MIT-metal ions transport.

To further explore the functions of the two sets of lineage-specific genes within zebrafish, their cellular roles and GO categories were predicted using the ProtFun 2.2 server (Figure 3). As with the orphan genes in mice, orphan genes and CTSGs within zebrafish that were functionally associated with translation and energy metabolism were the most commonly presented, followed by those associated with transport, binding, and regulatory functions [69]. Furthermore, amino acid biosynthesis, replication, transcription, and the cell envelope were well represented compared with other cellular categories such as the biosynthesis of cofactors, cellular processes, purines, and pyrimidines. The percentages of orphan genes and CTSGs with the same cellular roles were generally similar except one; specifically, about 33% of CTSGs were associated with the cell envelope, which was significantly higher than that of the orphan genes (4%). With respect to the GO categories predicted by ProtFun, about 70% and 80% of orphan genes and CTSGs, respectively, were able to be assigned to a GO category. The growth factor category (about 25%) was a relatively abundant category for both orphan genes and CTSGs. Strikingly and interestingly, the proteins of CTSGs involved in immune response were the most represented, exhibiting a percentage of 30.65%, which is approximately two times that of the same GO category for the orphan genes, implying a significantly larger expansion of these genes in teleosts. In contrast, the proteins of orphan genes involved in transcription regulation were the second most represented (23%), exhibiting a percentage about three times more than that of the same category of proteins in CTSGs. Although there was a remarkable difference in the percentage, the numbers of both orphan genes and CTSGs of this GO category were very similar (11 and 9, respectively). In addition, the proteins associated with signal transducer, receptor, hormone, structural protein, and stress response were well represented compared with the GO category of ion channel proteins, which included associations to the ion channel, voltage-gated ion channel, cation channel, and metal ion transport. These functional data suggest that lineage-specific genes may indeed be excellent candidates for genes involved in important housekeeping functions in a specific clade or species [36,40].

### High percentage of lineage-specific genes generated via gene duplications

Gene duplication followed by rapid sequence divergence by a paralog, which is beyond the threshold of similarity searches, has long been thought to be the major mechanism that provided raw materials for the emergence of new genes since the publication of the famous monograph authored by Susumu Ohno [70], although there are several other hypotheses regarding the origin of the lineage-specific genes, such as horizontal gene transfer [71,72], an accelerated evolutionary rate [50], de novo emergence from non-genic sequences [73] as well as artifacts from genome annotation [42]. In order to determine the proportion of contribution by gene duplication to the lineage-specific genes within zebrafish, we sought to identify such lineage-specific genes generated from the duplication-divergence mechanism using a simple method; that is, we determined whether any paralogs of the lineage-specific genes were widely evolutionarily conserved [34]. To ascertain a minimum percentage of genes that may be generated via gene doubling, we first downloaded the information regarding the paralogs of CTSGs and orphan genes annotated in Ensembl version 64 using Biomart. Secondly, paralogs that were also found to be evolutionarily conserved were considered to be associated with lineage-specific genes created via gene duplication followed by rapid sequence divergence.

**Figure 4:**
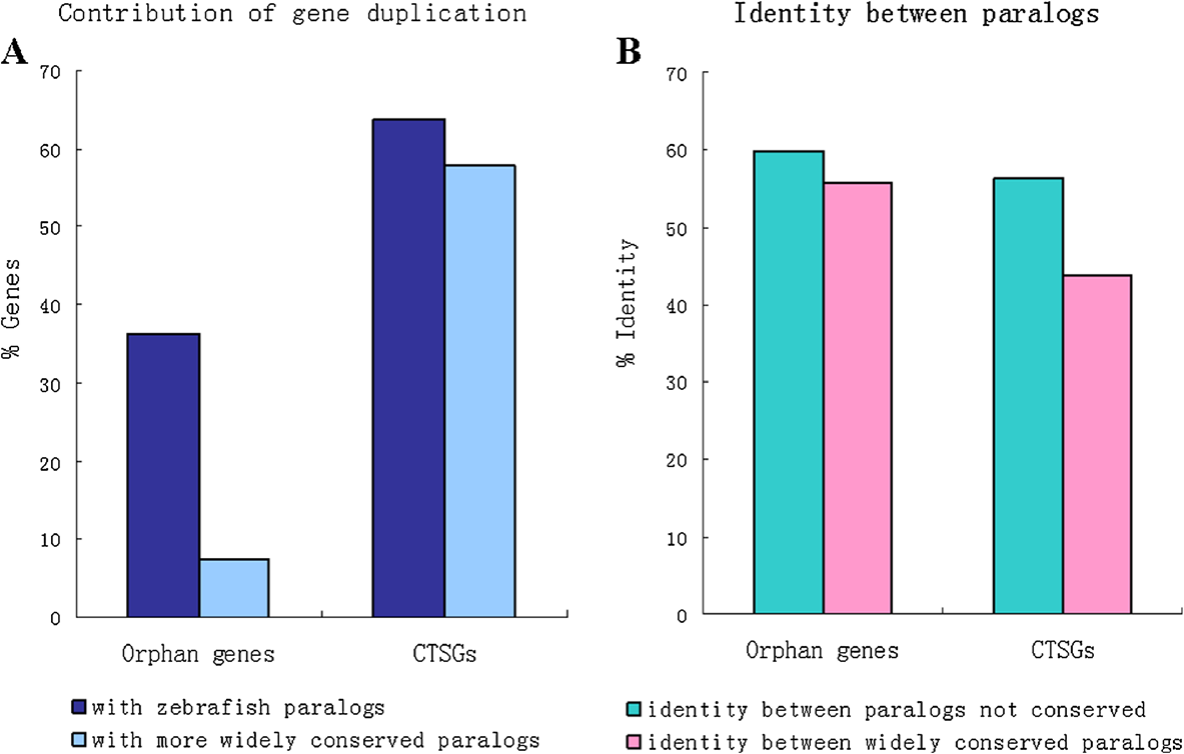
Percentages of lineage-specific genes within zebrafish with evidence for origin by gene duplication (A) and similarity between lineage-specific genes and their widely evolutionary conserved paralogs as well as not widely evolutionary conserved paralogs (B).

Through this analysis, we found about 36.4% (24) of orphan genes had paralogs, of which only five (7.6%) were evolutionarily conserved. That is, only about 7.6% of orphan genes were generated via gene doubling. Compared with the relatively low percentage of orphan genes formed by gene duplication, the contribution of gene duplication to the generation of CTSGs was very remarkable. As much as 57.8% of CTSGs could have emerged as a result of the duplication-divergence mechanism, which was much higher than previous reports for insects [40] and primate [34], but lower than reports for Drosophila [74]. In addition, if a lineage-specific gene is generated by gene duplication followed by rapid sequence divergence, then the similarity between the lineage-specific gene and its evolutionarily conserved paralogs should be lower than that between the lineage-specific gene and its evolutionarily not conserved paralogs. To test this hypothesis, we further explored the similarity between the lineage-specific genes and their paralogs, both evolutionarily conserved and not evolutionarily conserved. Consistent with the hypothesis, the average similarity of CTSGs with their evolutionarily conserved paralogs was significantly lower than their average similarity with the other group of paralogs (*t*-test, *p* < 1e-3). However, there was no significant difference between orphan genes and their paralogs (Figure 4). These data further confirm that gene duplication followed by rapid sequence divergence was a major mechanism generating new genes [75]. Lists of all orphan genes and CTSGs with putative parent genes in the zebrafish genome that are widely conserved as well as not conserved are provided (Additional file 3: Table S4 and Additional file 4: Table S5).

### Orphan genes are preferentially expressed in the reproductive system

The EST database is a collection of millions of ESTs gathered from thousands of RNA libraries covering dozens of zebrafish organs or tissues at different developmental stages [76]. In order to elucidate the expression patterns of the lineage-specific genes in zebrafish, we first analyzed this comprehensive dataset to detect whether the lineage-specific genes were preferentially expressed in certain tissues or organs. One key step in achieving this goal was the reliable mapping of EST and full-length cDNA to genomic sequences, so we followed a relatively stringent pipeline [77] to retain high quality mappings (see Methods).We counted a gene as expressed in a tissue or organ as long as it was supported by one EST.

Through this stringent pipeline, we found that orphan genes were preferentially expressed in reproductive organs and tissues compared with other organs at a 5% significance level (Table 2; Fisher’s Exact Test, *p* < 1e-3). The same test for the brain showed no significant enrichment of orphan genes, which was the same observation when analyzing primate-specific genes [39]. Nevertheless, there was no significant enrichment in gonad expression for the CTSGs. Considering that the EST database covers a large number of tissues and organs, these observations suggest that the orphan genes within zebrafish were preferentially enriched for expression of the reproductive system compared with the CTSGs. Our result is in accordance with the association of recent-origin genes with both reproductive expression [78] and reproduction-related behavior [79].

**Table 2:** Tissue distribution of expressed lineage-specific genes

**Type of genes**	**Gonad**	**Brain**	**Liver**	**Kidney**	**Sense organ**
Orphan genes	12(50.0 %)	1(4.2 %)	1(4.2 %)	2(8.3 %)	2(8.3 %)
CTSGs	17(19.5 %)	22(25.3 %)	13(14.9 %)	9(10.3 %)	19(21.8 %)

### Temporal and spatial expression profiles of lineage-specific genes

Knowing the expression patterns of lineage-specific genes at different developmental stages and in different tissues or organs is essential to illuminate whether the lineage-specific genes have corresponding biological function. Therefore, we exploited the RNA-seq data to assess a far more precise measurement of the temporal and spatial expression profiles of these lineage-specific genes. RNA-seq is a recently developed method to transcriptome profiling that uses high throughput technologies and is a powerful method to quantify the expression of genes [80]. A time-series of RNA-seq data from 15 time-points during early zebrafish organogenesis that mark important developmental stages was obtained from previous studies [81-83]. RNA-seq data from 5 different zebrafish tissues were downloaded from NCBI [84]. The RPKM, defined here as the number of unique mapped reads to the coding regions divided by one thousandth of the total length of all the exons of the gene, subsequently normalized by dividing by one millionth of the total number of valid reads, was calculated (see Methods).

Evidence for the expression of the lineage-specific genes in zebrafish, here defined as the mapped reads to their coding regions, was found in the transcriptome data for 54 of the 66 orphan genes (81.8%) and 129 of the 135 CTSGs (95.6%). These percentages were significantly higher than those observed using EST/FL-cDNA data. For the remaining 12 orphan genes not represented by RNA-seq data, one had evidence of expression in EST data and one had evidence of expression in protein data. Thus, the failure to find evidence for expression of these two genes with RNA-seq data may suggest that the two genes were expressed in other developmental stages and tissues or at a very low level in the analyzed developmental stages and tissues, or the expression evidence based on the EST and protein data may be incorrect (i.e. as a result of contamination by other samples). As for the other 10 orphan genes not represented by RNA-seq data, their gene models have probably been incorrectly annotated, considering that each of them had a transcript less than 60 bp. On the other hand, expression evidence for the 6 CTSGs not represented by RNA-seq data had all been found from EST data. Therefore, the failure to find evidence for expression using RNA-seq data may suggest that these genes were expressed in other developmental stages or tissues. For example, gene ENSDARG00000094271 was expressed in olfactory tissue at about 3–4 months old and gene ENSDARG00000089157 was expressed in kidney tissue, which was not included in our RNA-seq data.

**Figure 5:**
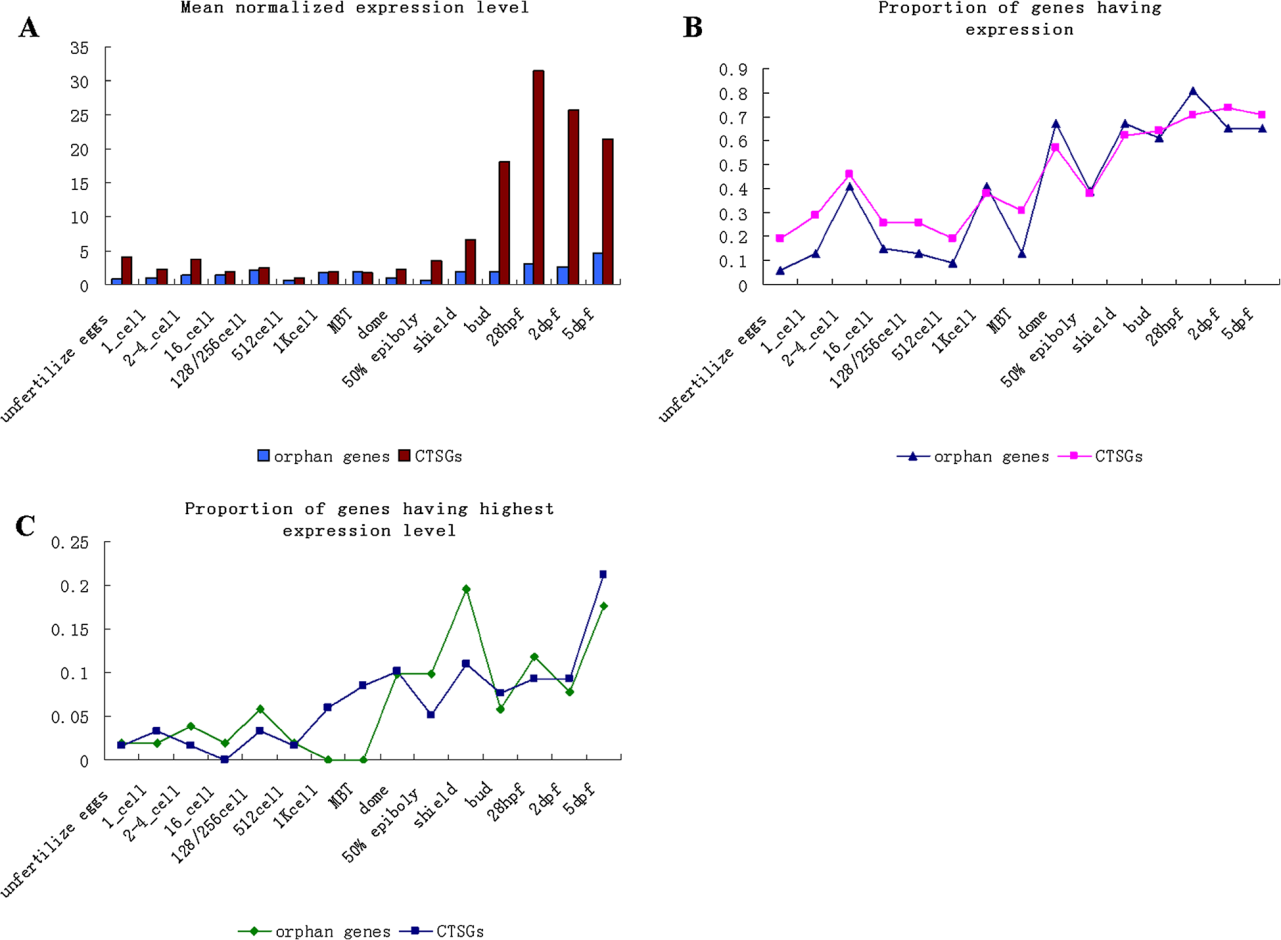
**Temporal expression profiles of lineage-specific genes during zebrafish embryogenesis.** (**A**) Mean normalized expression levels of lineage-specific genes during embryogenesis are defined by the mean level of expression as the sum of all the RPKM for each developmental stages divided by the total number of lineage-specific genes. The vertical axis represents value of mean the normalized expression levels and abscissa axis represents the 15 time-points. (**B**) The proportion of the lineage-specific genes that have expressed reads in the 15 time-points. (**C**) The proportion of the lineage-specific genes having their highest normalized expression levels in each of the 15 developmental stages.

In addition to the high tissue preferences observed in previous studies, we also discovered two intriguing temporal expression patterns of the lineage-specific genes within zebrafish. First, lineage-specific genes were more likely to be enriched in later-stage embryos and early larval stages with a higher fraction of orphan genes. The mean normalized level of gene expression, as defined here by the sum of all the RPKM for all developmental stages divided by the total number of lineage-specific genes, was generally higher in later-stage embryos and early larval stages than in early-stage embryos (Figure 5A). On the other hand, a larger proportion of the lineage-specific genes were expressed during the later-stage embryos and early larval stages than during the early-stage embryos (Figure 5B). Interestingly, upon sorting the normalized expression levels of the lineage-specific genes for the 15 time-points from highest to lowest, we found that the proportion of genes having the highest expression level in the developmental stage, which was defined as the numbers of genes having highest expression level in the developmental stage divided by total gene number, was also higher in later-stage embryos and early larval stages than in early-stage embryos (Figure 5C). A similar pattern was also observed for the proportion of genes exhibiting the second, third, and fourth highest levels of expression (Additional file 5: Figure S1). Finally, the percentage of high expression level in later-stage embryos and early larval stages was higher for orphan genes compared to the CTSGs (25 versus 29 or 37.9% versus 21.5%, Chi-square test, *p* < 0.05). Collectively, these results strongly suggest that lineage-specific genes were more preferentially to be enriched in later-stage embryos and early larval stages. Second, we calculated the Shannon entropy-based specificity score per genes as a measure of expression level divergence during zebrafish embryogenesis (see Methods) to test whether there were more pronounced changes in gene expression level between two consecutive developmental stages for lineage-specific genes than for evolutionarily conserved genes. We found that the entire two sets of lineage-specific genes showed an increased temporal specificity compared with the evolutionarily conserved genes (one-way ANOVA; *p* < 10^-3^). This phenomenon suggests that lineage-specific genes had a more restricted temporal expression than the EC genes. In addition, RNA-seq data showed that orphan genes were preferentially expressed in the ovary and CTSGs were enriched for brain expression. The tissue that had the largest proportion of the genes expressed and the proportion of genes exhibiting the highest expression level in the tissue basically support the above conclusion, which is also consistent with the evidence from the EST data.

Several individual lineage-specific genes were also found to have intriguing expression patterns (Additional file 6: Figure S2). For example, orphan gene ENSDARG00000095794 exhibited significantly high expression in the ovary; thus, we speculated that that gene may play a role in reproduction. Orphan gene ENSDARG00000090169 was highly, and nearly specifically, expressed in the post-MBT stages, suggesting that this gene may contribute to the development of later-stage embryos and early larval stages. One of CTSGs, ENSDARG00000076244, was highly expressed in both female and male brains, which indicates that this gene may have a role in zebrafish brain development. Another CTSG, ENSDARG00000017163, exhibited significantly high expression in the early-stage embryos, elucidating its important contribution to the development of early embryos development. A list of the number of unique mapped reads and the RPKM of each lineage-specific gene is provided (Additional file 7: Table S6_1 and Table S6_2).

In order to validate the expression pattern of these lineage-specific genes, reverse transcription polymerase chain reaction (RT-PCR) assay was used. Primers for 16 lineage-specific genes (5 orphan genes and 11 CTSGs) were designed and all of these genes were amplified. The information of the primers and the results of RT-PCR were provided (Additional file 8: Table S7 and Additional file 9: Figure S3).

## Discussion

Enormous lineage-specific genes identified in other taxa with potentially important functions [36-41,55,60,85,86] motivated our genomewide search for lineage-specific genes within zebrafish. Here, we adopted BLAST [62], the preferred method for detecting homologs, and phylostratigraphy [61] to identify two sets of lineage-specific genes within zebrafish. Then we characterized these genes, predicted their functions *ab initio*, inferred their evolutionary origin, and analyzed their expression patterns, making this the most comprehensive study of lineage-specific genes within teleosts and zebrafish to date. The 135 CTSGs and 66 orphan genes obtained in this study are attractive targets for future experimental discovery, owing to their lineage specificity and to the fact that the majority encode proteins whose functions are yet to be determined (while only one orphan gene and 42 CTSGs have GO term accession). Compared with the lineage-specific genes identified in plants [36,43,44], the number of lineage-specific genes within zebrafish is significantly lower, which may reflect the basic difference between animals and plants, considering the likely small number of lineage-specific genes identified in primate [39] and insects [40,41]. Although Yang *et al.*[60] identified a relatively small number of lineage-specific genes in Arabidopsis, Oryza, and Populus, whose number is close to that in animals, their criteria used to define sequence conservation was too relaxed, making the validity of their results questionionable. For example, they restricted their analysis to only the genes with expression evidence support and employed a very relaxed criterion to define sequence conservation (e-value cutoff of 0.1) that has not been used in other studies. Taken together, the dramatic difference in number of lineage-specific genes observed between the genomes of animals and plants should not be the result of the method we used and may suggest that there is a remarkable genetic difference in terms of lineage-specific genes between the genomes of animals and plants. In addition, this difference may suggest that genome doubling followed by sequence divergence occurred in plants at a higher frequency [87], which may explain to some extent why there are many more lineage-specific genes in plant genomes.

Both the CTSGs and orphan genes had shorter gene length compared with the EC genes, probably owing to fewer numbers of exons per gene and higher percentage of intronless lineage-specific genes. For example, nearly 28% of orphan genes contained only one exon, while the percentage of single exon EC genes was only 6%. One reason for such a difference may be that intronless genes can arise via retroposition, which has been confirmed to create a large amount of new genes in the zebrafish genome [88]. Alternatively, this difference may be a result of the “introns late” hypothesis, which assumes intron accretion into the protein-coding genes is continuous throughout the evolutionary time of eukaryotes [89]. Thus, the younger the genes are, the fewer exons they have. Additionally, since orphan genes are species specific, these genes may have arisen in relatively recent years. Collectively, these reasons may partly explain why young orphan genes contain a single exon and why lineage-specific genes are shorter than older evolutionarily conserved genes.

Generally speaking, lineage-specific genes are thought to play significant roles in the evolution of lineage specific phenotypes and adaptive innovation [90]. Although there are a large number of lineage-specific genes whose functions have not been characterized and only one orphan gene and 31% CTSGs have GO term accession, we were still able to find five orphan genes and eight CTSGs whose functions are closely related to immunity. The significant enrichment of immunity proteins in the lineage-specific genes within zebrafish indicates that defense against pathogens may be an important goal in terms of the successful diversification of fishes. Fishes are an extremely diverse group of aquatic vertebrate animals that also exhibit enormous diversity in the habitats they occupy. Fishes live in almost every conceivable type of aquatic habitat, from an elevation up to 5,200 meters in Tibet to 7,000 meters below the surface of the ocean and some species even make short excursions onto land. Some fishes can also live in almost pure freshwater, while others reside in very salty lakes. They can tolerate temperatures ranging from as high as 42.5°C to −2°C under the Antarctic ice sheet [1]. Thus, fishes should be confronted with much more diverse pathogen invasion. Therefore, lineage-specific genes involved with immunity should help fishes better adapt to various pathogens and successfully survive within their diverse habitats. In addition, the prediction of gene function is based on homology to proteins with known function in other species. Some lineage-specific genes lack homologs in other lineages, so we predicted their function *ab initio*. Interestingly, the proteins of CTSGs involved in immune response were the most represented, with a percentage of 30.65% of CTSGs, probably implying a significantly larger expansion of these genes in teleosts. Therefore, function assignment both based on homology and prediction *ab initio* showed a significant enrichment in proteins related to immune response, suggesting that the successful adaptation of teleosts may be explained by their conserved lineage-specific genes.

Variation of gene number within different organisms suggests a general process of new gene origination [54]. One basic question in biology is the molecular mechanisms involved in the creation of new genes. There have already been several hypotheses regarding the origin of lineage-specific genes. However, determining the exact mechanisms regarding the origin of lineage-specific genes depends on the comparative genome analysis of taxonomically closely related species. It is extremely difficult to achieve the aforementioned goal for research on fish so far. Gene duplication followed by rapid sequence divergence in one of the paralogs is a well explored source of lineage-specific genes [75,91]. A simple method for determining such genes is to determine whether any of the paralogs of lineage-specific genes are widely evolutionarily conserved. Through this analysis, we found that there were a significantly large number of lineage-specific genes generated by gene duplication followed by rapid sequence divergence of one of the paralogs. It was also confirmed by observing that the similarity between the genes and their evolutionarily conserved paralogs was lower than the similarity between the genes and their paralogs not evolutionarily conserved. As for other mechanisms forming lineage-specific genes, we will explore these questions in the future when the genome sequence of the silver carp (*Hypophthalmichthys molitrix*), a relatively close species to zebrafish, is released.

Previous studies have shown that young new genes generated by various mechanisms seem to have been preferentially endowed with testis-specific or testis-biased expression patterns [90]. In accordance with this observation, there are a significantly large number of new genes within zebrafish expressed in the reproductive system reflecting the expectation that emergence of new, lineage-specific genes may accompany speciation or reproduction. This suggests that this expression pattern is a general phenomenon not only in mammals and *Drosophila*, but also in teleosts. There are several hypotheses which can explain this propensity. First, sex- and reproduction-related genes are generally recognized as a class of rapidly evolving genes and undergo adaptive evolution after speciation events involved in male reproduction [92]. Furthermore, the testis is the most rapidly evolving organ owing to the strong selective pressures to which it is subjected because of its important roles in sperm competition, sexual conflict, reproductive isolation, germline pathogens, and mutations causing segregation distortion in the male germline [90]. Second, the “hypertranscription” state [93] caused by chromatin remodelling and RNA polymerase II complexes in the meiotic and postmeiotic spermatogenic cells would favor the initial, unprovoked transcription of newly arisen genes [94]. As for the CTSGs, however, no significant reproductive expression was enriched, which further confirmed that only the young new genes were specifically expressed in the testis, since the CTSGs were relatively older than the orphan genes.

Expression analyses of lineage-specific genes using EST or microarrays have elucidated the fact that lineage-specific novel genes are preferentially expressed in specific tissues or organs, such as the testis or brain [39,90]. Although EST data covers a large number of samples, which could be used to compare the expression between different samples, the coverage of individual genes is too low to quantify the expression level of genes. Microarrays also have some limitations, such as cross-hybridization and saturation of signals [80]. Therefore, we used the RNA-seq data from various developmental stages and tissues to quantify the lineage-specific genes and highlight two novel properties of these genes. First, in addition to being highly tissue-specific, lineage-specific gene expression were highly temporally restricted. Second, lineage-specific genes were preferentially expressed in later-stage embryos and early larval stages compared with early-embryos. The higher expression level of lineage-specific genes after the MBT suggests that lineage-specific genes are important components for the zygotic transcription. Maternally deposited mRNAs direct early development before the initiation of zygotic transcription during mid-blastula transition [95]. However, zygotic transcription plays a more important role in the regulation of development after MBT, since a high percentage of maternally stored mRNA has been degraded during the post-MBT stages. In addition, it has been shown that all vertebrate embryos must converge towards a narrow point, called phylotypic stage at which all vertebrate show high morphogenetic resemblance, to acquire the basic scheme on which subsequent differences will emerge [96]. The phenomenon that more lineage-specific genes are expressed after the phylotypic stages may probably be linked to the acquisition of species-specific morphological traits. All vertebrate resemble each other at the phylotypic stage, so the crucial steps to form the morphological differences between species resulting from the expression product after the phylotypic stages. Therefore, lineage-specific genes within zebrafish should be crucial for the significantly morphological diversity of teleosts. On the other hand, Lineage-specific genes showed relatively higher expression levels during early larval stages, making them candidates for functions in specific tissues and organs during organogenesis. Expression analysis using RNA-seq from different tissues and organs supported the observations from the EST data and further showed that orphan genes are preferentially expressed in reproductive tissues, which also confirmed the potential roles of lineage-specific genes during organogenesis.

## Conclusions

In the study, we have identified two sets of lineage-specific genes, CTSGs and orphan genes, which are specific to teleosts and zebrafish, respectively. The Conserved Teleost-Specific Genes were found to be especially enriched in proteins with immunity functions, implying that defense against invasion by diverse pathogens was critical to the successful diversification of teleosts. We also revealed that, in addition to being highly tissue-preferred expression, lineage-specific genes are also highly temporally restricted and are preferentially expressed in later-stage embryos and early larval stages compared with early-embryos. This study provides valuable information for further analysis of the functions of these genes during zebrafish embryogenesis and will be helpful in improving the understanding of the successful diversification of teleosts.

## Methods

### Sequence data sets

Both the detection method and the reference set of genomes to be blasted are important for identifying lineage-specific genes, so we used a method called ‘phylostratigraphy’ to obtain the lineage-specific genes within zebrafish [32,61]. To identify CTSGs and zebrafish-specific genes (orphan genes), a total of 61 genomes and 59 proteomes were used in this study (Additional file 10: Table S1). Most of the proteomes and genomes data sets were downloaded from Ensembl version 64 [67], while the genome of *Salmo salar* was downloaded from NCBI [76]. The genome and protein sequences of *Branchiostoma floridae* were obtained from the website of the Joint Genome Institute (http://genome.jgi-psf.org/Brafl1/Brafl1.info.html). The genome of *Callorhinchus milii* was obtained from http://esharkgenome.imcb.a-star.edu.sg/. The protein data from UniProtKB was downloaded from UniProt ftp://ftp.ebi.ac.uk/pub/databases/uniprot/knowledgebase/. In all cases, the genomes and protein sets used were the latest versions.

### Homolog search

The two sets of lineage-specific genes within zebrafish were identified in a pipeline (Figure 1) based on a homolog search using BLASTp and tBLASTn, as well as BLASTx [62] with an e-value cutoff of 10^-5^[36,41,44]. We classified the zebrafish genes into three categories: Evolutionarily Conserved genes (ECs), CTSGs, and orphan genes. Here, orphan genes refer to genes for which we could not find homologs in any other species. CTSGs include genes for which we could find at least one homolog in teleosts, but no homologs anywhere else. ECs were genes with at least one homolog outside the group of teleosts.

### Gene characteristics and chromosomal localization

The genic information for the orphan genes, CTSGs, and ECs were downloaded from Ensembl version 64 using BIOMART (http://www.ensembl.org/). We then used Perl scripts to calculate gene length, protein length, number of exons, and GC content of the genes. We used one-way ANOVA to determine significant differences between the different sets of lineage-specific genes and the ECs. The chromosomal localization of each lineage-specific gene was also downloaded using BIOMART. In order to determine whether a gene had a transcript support, we used the results from the section “Expression analysis using EST and full-length cDNA.”

### Protein function assignment and category

Since there were few homologs between the lineage-specific genes and the genes in the public database, ProtFun 2.2 server [97,98] was employed to predict the cellular role and the gene ontology (GO) category of the entire two sets of lineage-specific genes. The prediction of cellular function and the GO category by ProtFun relies on a large number of other sequence derived protein features, including predicted post translational modifications (PTMs), protein sorting signals and physical/chemical properties, rather than relying on sequence similarity protein [97,98]. Therefore, ProtFun allows for the prediction of the function for even orphan proteins where no homolog can be found. Here, we used the ProtFun 2.2 server (http://www.cbs.dtu.dk/services/ProtFun/) to determine the functional categories of these two sets of lineage-specific genes and then clustered these sequences according to their cellular roles and GO categories.

### Gene duplication analysis

Gene duplication has long been thought as a major mechanism providing raw materials for the origin of new genes and innovations for genome evolution. Thus, we sought out to determine which lineage-specific genes had paralogs in the zebrafish that were more evolutionarily conserved than the lineage-specific genes. Such genes may indicate that the corresponding lineage-specific gene was generated via gene duplication followed by rapid sequence divergence. To accomplish this, we first downloaded the paralogs of orphan genes and CTSGs annotated in Ensembl using Biomart. Then, we further analyzed the lineage-specific genes that have paralogs to determine if any were more evolutionarily conserved; that is, at least one of the paralogs have homologs outside the teleosts or zebrafish.

### Expression analysis using EST and full-length cDNA

The expression data for EST and full-length cDNA (FL-cDNA) of zebrafish were downloaded from the UCSC (http://hgdownload.cse.ucsc.edu/downloads.html) [99]. EST and FL-cDNA data processing, such as genomic mapping, quality control for alignment, and EST or FL-cDNA to zebrafish gene mapping followed [77], which imposed a stringent quality control to retain high-quality mappings. First, we mapped the 1,488,275 EST sequences and 29,480 FL-cDNA to the zebrafish genome using BLAT [100] with the default parameters, which could eliminate sequences shorter than 100 bp. Then, we imposed the following criteria to discard low-quality mappings: mapping length ≥ 100 bp, identity ≥ 96%, coverage within mapping ≥ 97%, and coverage within whole transcript ≥ 75%. If a transcript was mapped to multiple genomic loci, then only the best mapping was retained; if more than one nearly identical best mapping existed (difference in BLAT scores < 5%), then the transcript was discarded to avoid ambiguity. Finally, only when a transcript overlapped with a gene longer than 100 bp and their directions were the same was that the transcript considered transcribed from the gene. These relatively stringent quality controls ensured the correct expression analysis. We counted a gene as expressed in a tissue as long as it was supported by only one EST. Then, we downloaded and extracted the tissue information for the expressed lineage-specific genes from NCBI using Batch Entrez (http://www.ncbi.nlm.nih.gov/sites/batchentrez/).

### Characterization of expression patterns for CTSGs and orphan genes by RNA–Seq

RNA-Seq, a recently developed method to transcriptome profiling that uses high-throughput sequencing technologies, has been shown to be extremely accurate for quantifying expression levels of genes and should have to revolutionize the manner in which eukaryotic transcriptomes are studied [80]. RNA-seq data from 4 zebrafish developmental stages: 1-cell [0.75 hour post fertilization (hpf)], 16-cell (1.5 hpf), 512-cell (2.75 hpf), and 50% epiboly (5.25 hpf) stages were obtained [81] and downloaded from NCBI with accession code ERP000635; from 6 zebrafish developmental stages: unfertilized eggs, 1-cell (~0.7 hpf), 16-cell stage (~1.5 hpf), 128-cell stage (~2.5 hpf), mid-blastula transition (MBT; ~3.5 hpf), and post-MBT (~5.3 hpf) from [82] with NCBI accession code GSE22830; from 8 zebrafish developmental stages: two to four cell, 1000 cell (3 hpf), dome (4.5 hpf), shield (6 hpf), bud (10 hpf), 28 hpf, 48 hpf, and 120 hpf were obtained and downloaded from NCBI [83] with accession code GSE32898. RNA-Seq data from 5 zebrafish tissues: adult zebrafish ovary, male adult zebrafish head, female adult zebrafish head, whole male adult zebrafish without head or testis, and whole female adult zebrafish without head or ovary were downloaded from NCBI with accession code ERP000016 [84]. We then calculated gene-level measurements, specifically reads per kilobase of exon model per million mapped reads (RPKM) following [101]. The RNA-seq data from the same developmental stages were put together. Briefly, we mapped all reads per time-point independently back to the zebrafish genome [Zv9] with TopHat (version 1.4.1) [102] and reads count per gene were calculated using htseq-count. Only reads that mapped to a unique location in the zebrafish genome were considered in the subsequent analyses. The expression level of a gene in a developmental stage or in a tissue was defined by the number of uniquely mapped reads in the gene divided by one thousandth of the whole exon length of the gene, then was normalized by dividing by one millionth of the total number of valid reads in the respect samples.

In order to evaluate the temporal specificity between the lineage-specific genes and the evolutionarily conserved genes, the temporal specificity score, here defined as 1- H(g)/log2(N), of the different sets of genes were determined, where H(g) is the Shannon entropy that could be a good measure of uncertainty.

### Statistical analysis

In this study, we used one-way ANOVA followed by a Duncan’s post hoc test (for equal variance) or Dunnett’s T3 test (for unequal variance) to test whether there were significant differences between the characteristics of lineage-specific genes and that of ECs, as well as among the temporal specificity scores of the three categories of genes analyzed. The Spearman’s correlation test was used to determine whether the number of lineage-specific genes on chromosomes correlated with the length of the chromosomes. The *t*-test was used to determine the similarity between lineage-specific genes and their paralogs. The Fisher’s exact test was used to test whether there was expression enriched in specific tissues. The Chi-square test was used to detect any significant difference between lineage-specific genes enriched in later-stage embryos and early larval stages.

## Competing interests

The authors declare that they have no competing interests.

## Authors’ contributions

LY developed the algorithm, carried out the analyses and drafted the manuscript. MZ and BF participated in algorithm development and the data analysis. SH designed and participated in the analysis. All authors read and approved the final manuscript.

## Supplementary Material

Additional file 1: Table S2 List of orphan genes identified in the study. Click here for file

Additional file 2: Table S3 List of conserved teleosts specific genes. Click here for file

Additional file 3: Table S4 Lineage-specific genes with paralogs that are conserved outside the taxa. Click here for file

Additional file 4: Table S5 Lineage-specific genes with paralogs that are not conserved outside the taxa. Click here for file

Additional file 5: Figure S1 Proportion of genes having second (A), third (B), and fourth (C) highest expression levels in each developmental stage. Click here for file

Additional file 6: Figure S2 Four genes with special expression patterns. Click here for file

Additional file 7: Table S6 List of the number of unique mapped reads and RPKM for both orphan genes and CTSGs expressed at different developmental stages and different organs. Click here for file

Additional file 8: Table S7 The information of the primers for RT-PCR. Click here for file

Additional file 9: Figure S3 The results of RT-PCR. The products of RT-PCR were sorted by their length. Click here for file

Additional file 10: Table S1 List of the genomes and proteomes used in this study. Click here for file
