# Supplementary figures and images for: Genome-wide identification, characterization, and expression analysis of lineage-specific genes within zebrafish
Source: BMC Genomics. 2013 Jan 31;14:65. doi: 10.1186/1471-2164-14-65 (PMC3599513; doi:10.1186/1471-2164-14-65)

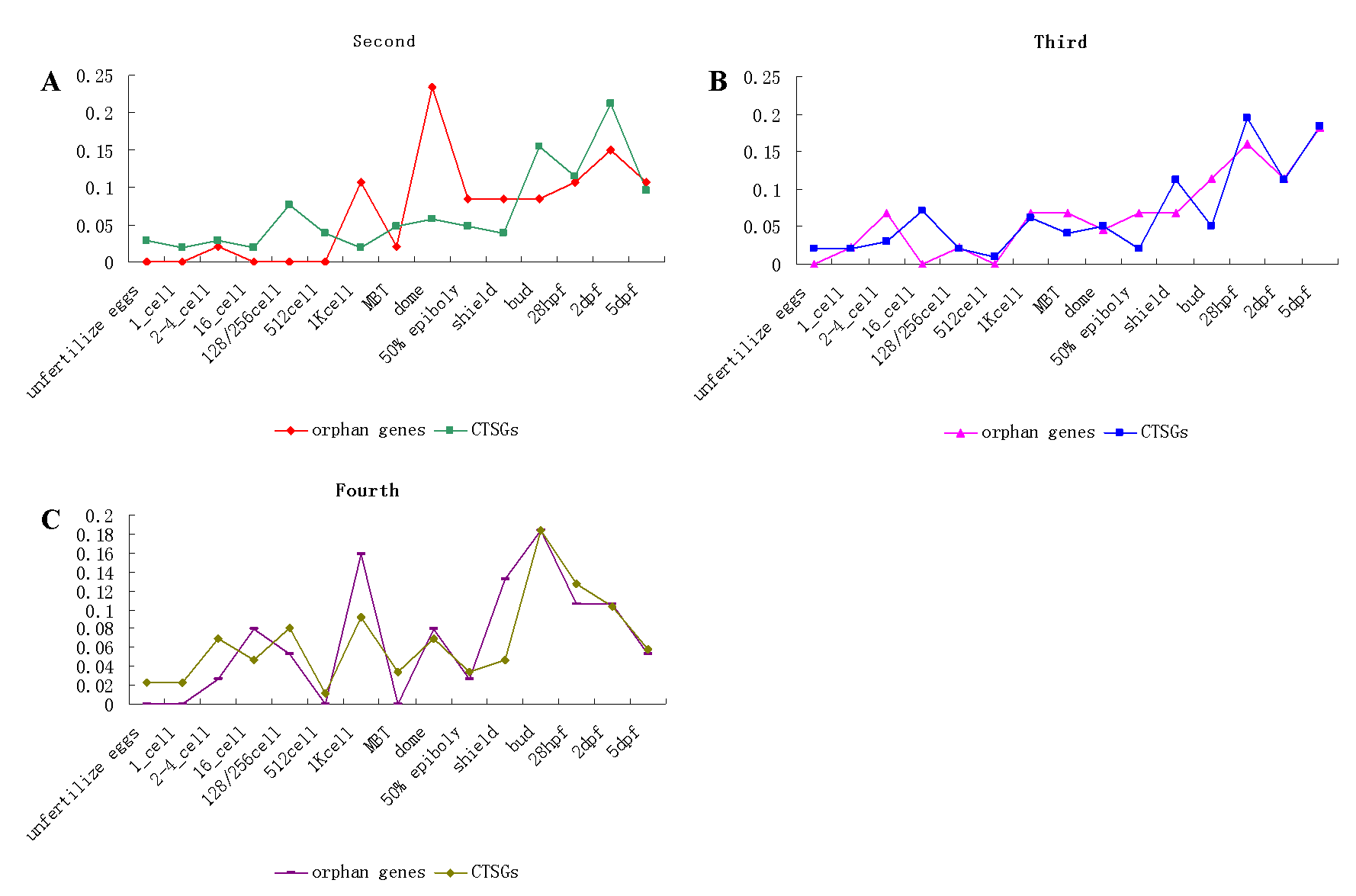

Supplement: Additional file 5: Figure S1 — Proportion of genes having second (A), third (B), and fourth (C) highest expression levels in each developmental stage. [file 1471-2164-14-65-S5.png]

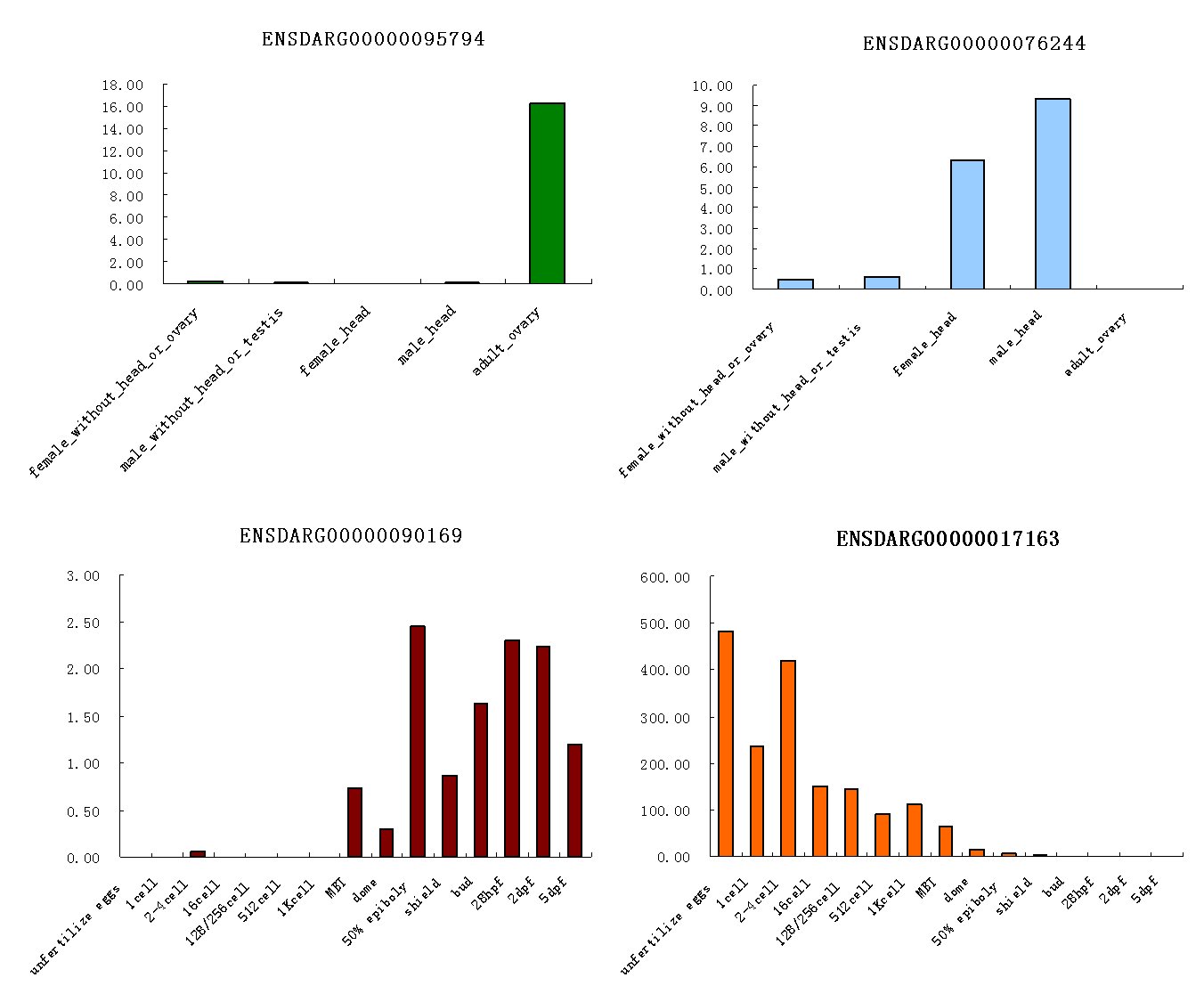

Supplement: Additional file 6: Figure S2 — Four genes with special expression patterns. [file 1471-2164-14-65-S6.png]

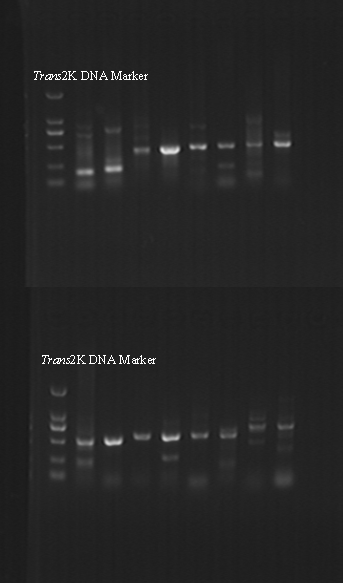

Supplement: Additional file 9: Figure S3 — The results of RT-PCR. The products of RT-PCR were sorted by their length. [file 1471-2164-14-65-S9.png]
